# Molecular Epidemiology and Functional Assessment of Novel Allelic Variants of *SLC26A4* in Non-Syndromic Hearing Loss Patients with Enlarged Vestibular Aqueduct in China

**DOI:** 10.1371/journal.pone.0049984

**Published:** 2012-11-21

**Authors:** Yongyi Yuan, Weiwei Guo, Jie Tang, Guozheng Zhang, Guojian Wang, Mingyu Han, Xun Zhang, Shiming Yang, David Z. Z. He, Pu Dai

**Affiliations:** 1 Department of Otolaryngology, Chinese PLA General Hospital, Beijing, People’s Republic of China; 2 Department of Biomedical Sciences, Creighton University School of Medicine, Omaha, Nebraska, United States of America; 3 Department of Otolaryngology, 3rd hospital of Hebei Medical University, Shijiazhuang, Hebei Province, People’s Republic of China; 4 Department of Otolaryngology, Hainan Branch of Chinese PLA General Hospital, Sanya, People’s Republic of China; Stanford University School of Medicine, United States of America

## Abstract

**Background:**

Mutations in *SLC26A4*, which encodes pendrin, are a common cause of deafness. *SLC26A4* mutations are responsible for Pendred syndrome and non-syndromic enlarged vestibular aqueduct (EVA). The mutation spectrum of *SLC26A4* varies widely among ethnic groups. To investigate the incidence of EVA in Chinese population and to provide appropriate genetic testing and counseling to patients with *SLC26A4* variants, we conducted a large-scale molecular epidemiological survey of *SLC26A4*.

**Methods:**

A total of 2352 unrelated non-syndromic hearing loss patients from 27 different regions of China were included. Hot spot regions of *SLC26A*4, exons 8, 10 and 19 were sequenced. For patients with one allelic variant in the hot spot regions, the other exons were sequenced one by one until two mutant alleles had been identified. Patients with *SLC26A4* variants were then examined by temporal bone computed tomography scan for radiological diagnosis of EVA. Ten *SLC26A4* variants were cloned for functional study. Confocal microscopy and radioisotope techniques were used to examine the membrane expression of pendrin and transporter function.

**Results:**

Of the 86 types of variants found, 47 have never been reported. The ratio of EVA in the Chinese deaf population was at least 11%, and that in patients of Han ethnicity reached at least 13%. The mutational spectrum and mutation detection rate of *SLC26A4* are distinct among both ethnicities and regions of Mainland China. Most of the variants caused retention of pendrin in the intracellular region. All the mutant pendrins showed significantly reduced transport capability.

**Conclusion:**

An overall description of the molecular epidemiological findings of *SLC26A4* in China is provided. The functional assessment procedure can be applied to identification of pathogenicity of variants. These findings are valuable for genetic diagnosis, genetic counseling, prenatal testing and pre-implantation diagnosis in EVA families.

## Introduction

*SLC26A4* encodes an anion transporter transmembrane protein, pendrin, which is expressed in the thyroid, kidney, and cochlea [1]. Pendrin is a member of the anion transporter family SLC26, which mediates the exchange of anions including Cl^-^, HCO_3_^-^, OH^-^, I^-^, or formate [2]. In thyrocytes, iodide and sodium are brought into the cells via the basolaterally located sodium–iodide symporter. Apically located pendrin seems to be responsible for the efflux of iodide into the follicular lumen [3]. In the kidney, pendrin is suspected to mediate Cl^−^/HCO_3_^-^ exchange in the acid–base regulating B- and non-A–non-B-intercalated cells [4]. Similarly, in the inner ear, pendrin is thought to mediate Cl^−^/HCO_3_^-^ exchange, and is therefore involved in the conditioning of endolymphatic fluid, presumably due to HCO_3_^-^ secretion [5]. Malfunction of pendrin leads to Pendred syndrome (PS) and non-syndromic DFNB4 deafness with EVA [6].

Pendred syndrome (PS) is the most common form of syndromic deafness and accounts for about 10% of hereditary hearing impairment [7]. It is an autosomal recessive disorder caused by biallelic mutations in *SLC26A4* resulting in hearing loss, enlargement of the vestibular aqueduct (EVA) and iodine organification defect in the thyroid gland [8]. EVA, diagnosed based on the criteria of a greater than 1.5-mm diameter at the midpoint between the common crus and the external aperture, is detected in the ears of patients with PS by computed tomography (CT) and magnetic resonance imaging (MRI) [9]. EVA is the most common form of inner ear malformations associated with prelingual or postlingual sensorineural hearing loss, and is an important feature of PS [10]. EVA may occur alone or in combination with an incomplete partition of the apical turn of the cochlea as part of a Mondini deformity. PS is differentiated from non-syndromic hearing loss with EVA by the presence of goiter, which usually develops later at around the time of puberty. Since environmental and other genetic factors may modulate the effects of *SLC26A4* mutations on the development of goiter, the expression of goiter in PS patients is variable and may have incomplete penetrance [11]. Hearing loss in PS and DFNB4 occurs either congenitally or after some mild head injury, showing fluctuating and progressive hearing loss.

To-date, 174 *SLC26A4* mutations have been reported [12]. The mutation spectrum varies widely among ethnic groups [8], [11], [13]–[21]. Park and Pryor observed that patients with PS were always associated with two mutant alleles in *SLC26A4*, consistent with autosomal recessive disorder, whereas patients with non-syndromic hearing loss and EVA might have one or no mutant allele [8], [11]. In a Caucasian non-syndromic EVA cohort, about one-third of the patients had two mutant alleles, one-third had one mutant allele and the remaining one-third had zero [11]. In Japanese and Korean, the proportions of EVA patients with two identified mutant alleles in *SLC26A4* are 57% and 81%, respectively [14], [20]. In China, 97.9% of the EVA patients in simplex families were detected with either biallelic or monoallelic mutations, of which 88.4% carried biallelic variants and 9.5% had a monoallelic mutation. Only 2.1% of the Chinese patients with EVA had no mutant *SLC26A4* allele detected [17].

*SLC26A4* mutations are the second-most common cause of deafness in China. The incidence of non-syndromic EVA accounts for 13.73% of hereditary hearing loss [22]. However, Pendred syndrome is relatively rare in the Chinese population. Molecular epidemiology surveys showed that there are more than 100 *SLC26A4* variants in the Chinese hearing loss population, of which at least half are novel. With genetic testing of deafness becoming more popular in China, verification of the pathogenicity of novel variants is essential for both genetic and prenatal diagnosis. However, the two parameters used thus far–(i) low incidence of the mutation in the control population and (ii) substitution of evolutionarily conserved amino acids by the mutation–are not sufficient for clinical genetic diagnosis. To investigate the incidence of EVA in Chinese hearing loss populations and provide appropriate genetic testing and counseling to patients with *SLC26A4* variants, we conducted a large-scale molecular epidemiological survey of *SLC26A4* in China. In addition, in order to assess the pathogenicity and understand the molecular mechanism(s) underlying EVA and hearing loss, we examined the membrane expression of pendrin and the transporter function of 10 variants that comprised six novel variants and four reported only in Chinese patients.

## Materials and Methods

### Subjects

A total of 2352 unrelated non-syndromic hearing loss patients from 27 different regions of China were included in this study. To sample as many regions of China as possible, we included the remote northwestern provinces, including Xinjiang (translates as ‘New Territory’), Tibet, and Qinghai, where minorities make up a significant proportion of the local population. We also included samples from the southwestern provinces of Yunnan and Guizhou, where the populations comprise a number of minorities originating from various native tribes. The patients consisted of 1314 males and 1038 females ranging in age from 1.8 to 24 years with an average age of 14.17±3.41. The majority of patients were Han Chinese (1903), followed by minorities in the Southwest region (125), Tibetan (119), Hui (89), minorities in Xinjiang (69), Mongolian (33), Maan (12) and Korean (2). Ethnic subgroup designations were based on permanent residency documentation.

The subjects in this study were from special educational schools within each region. All patients showed moderate to profound bilateral sensorineural hearing impairment on audiograms. Careful medical examinations revealed no clinical features other than hearing impairment. In addition to the 2352 patients, 200 Han individuals with normal hearing (87 females and 113 males, aged 15 to 30 years) were recruited as control subjects from Beijing (Northern) and Jiangsu Province (Eastern), two densely populated regions consisting of 98 to 99% Han Chinese. Auditory function was evaluated by pure-tone air-and bone-conduction threshold audiometry.

Written informed consent was obtained from all subjects or guardians prior to blood sampling and genetic testing. The study protocol including the consent procedure was performed with the approval of the Ethics Committee of the Chinese PLA General Hospital.

### Mutational Analysis of SLC26A4

DNA was extracted from peripheral blood leukocytes using a commercially available DNA extraction kit (Watson Biotechnologies Inc., Shanghai, China). The open reading frame (ORF) of *SLC26A4* includes 2–20 exons. DNA sequence analyses of *SLC26A4* were performed by polymerase chain reaction (PCR) amplification of the coding exons plus approximately 50–100 bp of the flanking intron regions followed by Big Dye sequencing and analysis using an ABI 3100 DNA sequencing machine (ABI, Foster City, USA.) and ABI 3100 Analysis Software v.3.7 NT, according to the manufacturer’s instructions. Because about 90% of Chinese EVA patients carried at least one allelic mutation in exons 8, 19 or 10 of *SLC26A4* [17], [18], [23], we designated the three exons as hot spot regions and sequenced these first in all 2352 patients. For the patients with one allelic mutation or variant in exons 8, 19 or 10, the other exons of *SLC26A4* were sequenced one by one until two mutant alleles had been identified.

Normal-hearing controls were screened in exons 2–21 of *SLC26A4* for mutations or variants by DHPLC followed by sequencing analysis.

### CT Scan and MRI

Patients with mutations or variants in *SLC26A4* were examined by temporal bone CT scan for diagnosis of inner ear malformation or EVA based on a diameter of >1.5 mm at the midpoint between the common crus and the external aperture. Some patients with EVA were further examined using magnetic resonance imaging (MRI).

### Pendrin cDNA and Site-directed Mutagenesis

The coding region of pendrin cDNA was amplified by PCR from the cDNA library of human thyroid tissue and subcloned into the pEGFP-N1 vector (Invitrogen, Carlsbad, CA, USA) using the *Xho*I and *Kpn*I restriction sites. The pendrin cDNA sequence was verified by nucleotide sequencing, and was identical to a registered sequence (GenBank NM_000441). Oligonucleotide directed mutagenesis was performed to create constructs of 10 mutant plasmids using the QuikChange® Site-Directed Mutagenesis Kit (Stratagene, La Jolla, California, USA), according to the manufacturer’s protocol. The cloning and mutagenic primers used in this study are listed in Table S1.

### Cell Culture and Transient Transfection

Human embryonic kidney (HEK) cells were cultured in DMEM solution (Invitrogen, Carlsbad, CA, USA) supplemented with 10% fetal bovine serum. Constructs of pendrin and its mutated plasmids were introduced to the cells using lipofectamine 2000 (Invitrogen, Carlsbad, CA, USA). The amount of DNA used per 35-mm dish was 4 µg, mixed with 10-µl lipofectamine. For radioisotope uptake experiments, the cells were passaged into 24-well plates 24-h before transfection, at 2×10^5^ cells per well. Cells were quantified using a hemocytometer (Fisher Scientific Inc., Pittsburgh, PA). The quantity of DNA used per well was 0.8 µg, added to 1.6 µl lipofectamine. Fluorescence microscopy was used to evaluate membrane targeting during cell-selection experiments.

### Transport Function Assessment

Conventional radioisotope techniques were used to measure transport functions from HEK cells transfected with plasmids encoding for pendrin and its mutated products, as described in previous studies [24]–[26]. Pendrin cDNA- and pEGFP-transfected cells were used as the positive and negative controls, respectively. To improve sensitivity, we measured transport function only from those cells that were positively transfected. Fluorescence-based flow cytometry was used for cell sorting. Cells with a fluorescence intensity 4–200 times greater than non-fluorescent cells were selected. In total, 600,000 events (cells) were collected for each sample. To measure [^14^C] formate uptake, sorted cells in the 24-well culture cluster were first incubated for 30 min in a solution containing 130 mM NaCl, 20 mM HEPES, 5 mM KCl, 5 mM glucose, 2 mM CaCl_2_ and 1 mM MgCl_2_ (pH 7.3 and 305 Osm/L). Cells were then incubated at RT for 12 min in a solution containing 140 mM K-gluconate, 20 mM HEPES and 5 mM glucose (pH 7.3 and 305 Osm/L). [^14^C] formate (Moravek Biochemicals, Inc., Brea, CA) was added to this solution at a concentration of 20 µM. Cells were then washed three times with cold K-gluconate solution without [^14^C] formate, lysed with 200 µl 0.5 M NaOH, and neutralized with 0.5 M HCl. The lysate was used for liquid scintillation counting to determine the [^14^C] formate uptake. In each run, three wells were assayed for each plasmid, and the experiments were repeated three times independently. Therefore, the data in each group represented a sample size (*n*) of 3×3 = 9 trials for each plasmid and control. Student’s *t*-test was used for statistical analysis.

## Results

By sequencing exons 8, 10, and 19 of *SLC26A4* in 2352 patients, we identified 342 patients with mutations or variants, of which 116 were homozygotes and 226 were heterozygotes. For the 226 patients with monoallelic mutations or variants in exons 8, 10, or 19 of *SLC26A4*, 132 were verified as compound heterozygotes by the subsequent whole ORF analysis, and the other 94 were verified as heterozygotes. The total variant detection rate was 14.54% (342/2352), including a homozygous mutation detection rate of 4.93% (116/2352), a compound heterozygous mutation detection rate of 5.44% (128/2352), and a monoallelic mutation detection rate of 4.17% (98/2352). For patients of Han ethnicity, accounting for 80.52% of our sample, the total variant detection rate, homozygous mutation detection rate, compound heterozygous mutation detection rate and monoallelic mutation detection rates were 16.55% (315/1903), 5.68% (108/1903), 6.46% (123/1903) and 4.41% (84/1903), respectively. Eighty-six types of variants in *SLC26A4* were found, including 7 nonsense variants, 13 frameshift variants, 48 missense variants, 5 splicing site variants, 6 silent variants, 1 untranslated region (UTR) variant and 6 intron variants (Table 1). The missense variants accounted for 60.76% (48/79, excluding the UTR variant and intron variants) of all the *SLC26A4* variants. Of the 86 variants, 47 were novel, which comprised 4 nonsense variants, 6 frameshift variants, 22 missense variants, 2 splicing site variants, 6 silent variants, 1 UTR variant and 6 intron variants (Table 2).

**Table 1:** Variants information of *SLC26A4* in Chinese population.

No.	Variants	Amino acid	Exon	Mutant allele frequency	Allele frequency	Number of patients
1	IVS7-2A>G		Spicing site	61.6%(**377**/612)	8.01%(377/4704)	271
2	c.2168A>G	p.H723R	19	11.60%(**71**/612)	1.51%(71/4704)	59
3	c.1174A>T	p.N392Y	10	2.12%(**13**/612)	0.28%(13/4704)	13
4	c.1229C>T	p.T410M	10	1.96%(**12**/612)	0.25%(12/4704)	12
5	c.2027T>A	p.L676Q	17	1.47%(**9**/612)	0.19%(9/4704)	9
6	15+5G>A		Spicing site	1.14%(**7**/612)	0.15%(7/4704)	8
7	c.589G>A	p.G197R	5	0.98%(**6**/612)	0.13%(6/4704)	6
8	c.1238A>G	p.Q413R	10	0.98%(**6**/612)	0.13%(6/4704)	6
9	c.1826T>G	p.V609G	17	0.82%(**5**/612)	0.11%(5/4704)	5
10	c.281C>T	p.T94I	3	0.65%(**4**/612)	0.08%(4/4704)	4
11	c.1226G>A	p.R409H	10	0.65%(**4**/612)	0.08%(4/4704)	4
12	c.1336C>T	p.Q446X	11	0.49%(**3**/612)	0.06%(3/4704)	3
13	c.1548insC	FS517,p.526X	14	0.49%(**3**/612)	0.06%(3/4704)	3
14	c.1975G>C	p.V659L	17	0.33%(**2**/612)	0.11%(5/4704)	2
15	c.259G>T	p.D87Y	3	0.33%(**2**/612)	0.04%(2/4704)	2
16	c.279T>A	p.S93R	3	0.33%(**2**/612)	0.04%(2/4704)	2
17	c.665G>T	p.G222V	6	0.33%(**2**/612)	0.04%(2/4704)	2
18	c.1079C>T	p.A360V	9	0.33%(**2**/612)	0.04%(2/4704)	2
19	c.1181_1183delTCT	p.S394del	10	0.33%(**2**/612)	0.04%(2/4704)	2
20	c.1225C>T	p.R409C	10	0.33%(**2**/612)	0.04%(2/4704)	2
21	c.1299_1300insC	FS434,p.467X	11	0.33%(**2**/612)	0.04%(2/4704)	2
22	c.1339_1340delA	FS447,p.454X	11	0.33%(**2**/612)	0.04%(2/4704)	2
23	c.1343C>A	p.S448X	12	0.33%(**2**/612)	0.04%(2/4704)	2
24	c.1595G>T	p.S532Y	14	0.33%(**2**/612)	0.04%(2/4704)	2
25	c.1687_1693insA	FS565,p.573X	15	0.33%(**2**/612)	0.04%(2/4704)	2
27	c.2162C>T	p.T721M	19	0.33%(**2**/612)	0.04%(2/4704)	2
28	c.2167C>G	p.H723D	19	0.33%(**2**/612)	0.04%(2/4704)	2
26	c.1985G>A	p.C662Y	17	0.16%(**1**/612)	0.04%(2/4704)	1
29	c.87G>C	p.E29D	2	0.16%(**1**/612)	0.02%(1/4704)	1
30	c.68C>A	p.S23X	2	0.16%(**1**/612)	0.02%(1/4704)	1
31	c.109G>T	p.E37X	2	0.16%(**1**/612)	0.02%(1/4704)	1
32	c.413_414delT	FS139,p.144X	4	0.16%(**1**/612)	0.02%(1/4704)	1
33	c.200C>G	p.T67S	3	0.16%(**1**/612)	0.02%(1/4704)	1
34	c.230A>T	p.K77I	3	0.16%(**1**/612)	0.02%(1/4704)	1
35	c.234_235delC	FS79,p.96X	3	0.16%(**1**/612)	0.02%(1/4704)	1
36	c.249G>A	p.W83X	3	0.16%(**1**/612)	0.02%(1/4704)	1
37	c.43_44insG	FS15,p.86X	2	0.16%(**1**/612)	0.02%(1/4704)	1
38	IVS4+2T>C		Spicing site	0.16%(**1**/612)	0.02%(1/4704)	1
39	IVS4+7A>G		Spicing site	0.16%(**1**/612)	0.02%(1/4704)	1
40	c.754T>C	p.S252P	6	0.16%(**1**/612)	0.02%(1/4704)	1
41	c.757A>G	p.I253V	6	0.16%(**1**/612)	0.02%(1/4704)	1
42	c.916_917insG	FS306,p.329X	7	0.16%(**1**/612)	0.02%(1/4704)	1
43	c.941C>T	p.S314L	8	0.16%(**1**/612)	0.02%(1/4704)	1
44	c.1019_1020delT	FS341,p.343X	9	0.16%(**1**/612)	0.02%(1/4704)	1
45	c.1124A>G	p.Y375C	9	0.16%(**1**/612)	0.02%(1/4704)	1
46	c.1173C>A	p.S391R	10	0.16%(**1**/612)	0.02%(1/4704)	1
47	c.1240G>A	p.E414K	10	0.16%(**1**/612)	0.02%(1/4704)	1
48	c.1245C>A	p.S415R	10	0.16%(**1**/612)	0.02%(1/4704)	1
49	c.1262A>C	p.Q421P	10	0.16%(**1**/612)	0.02%(1/4704)	1
50	c.1327G>C	p.E443Q	11	0.16%(**1**/612)	0.02%(1/4704)	1
51	c.1334T>G	p.L445W	11	0.16%(**1**/612)	0.02%(1/4704)	1
52	c.1409G>A	p.R470H	12	0.16%(**1**/612)	0.02%(1/4704)	1
53	c.1472T>C	p.I491T	13	0.16%(**1**/612)	0.02%(1/4704)	1
54	c.1517T>G	p.L506R	13	0.16%(**1**/612)	0.02%(1/4704)	1
55	c.1520delT	p.L597X	13	0.16%(**1**/612)	0.02%(1/4704)	1
56	IVS13+5G>A		Spicing site	0.16%(**1**/612)	0.02%(1/4704)	1
57	c.1586T>G	p.I529S	14	0.16%(**1**/612)	0.02%(1/4704)	1
58	c.1594A>C	p.S532R	14	0.16%(**1**/612)	0.02%(1/4704)	1
59	c.1645_1646insA	FS549,p.563X	15	0.16%(**1**/612)	0.02%(1/4704)	1
60	c.1667A>G	p.Y556C	15	0.16%(**1**/612)	0.02%(1/4704)	1
61	c.1678G>A	p.D560H	15	0.16%(**1**/612)	0.02%(1/4704)	1
62	c.1733_1735delATA	p.N579del.p.780X	16	0.16%(**1**/612)	0.02%(1/4704)	1
63	c.1790T>C	p.L597S	16	0.16%(**1**/612)	0.02%(1/4704)	1
64	c.1979T>G	p.L660R	17	0.16%(**1**/612)	0.02%(1/4704)	1
65	c.1988G>A	p.G663E	17	0.16%(**1**/612)	0.02%(1/4704)	1
66	c.1991C>T	p.A664V	17	0.16%(**1**/612)	0.02%(1/4704)	1
67	c.1897G>A	p.E633K	17	0.16%(**1**/612)	0.02%(1/4704)	1
68	c.1993A>G	p.I665V	17	0.16%(**1**/612)	0.02%(1/4704)	1
69	c.1983C>A	p.D661E	17	0.16%(**1**/612)	0.02%(1/4704)	1
70	c.2044G>T	p.E682X	18	0.16%(**1**/612)	0.02%(1/4704)	1
71	c.2228T>C	p.L743X	19	0.16%(**1**/612)	0.02%(1/4704)	1
72	c.2326C>G	p.R776G	21	0.16%(**1**/612)	0.02%(1/4704)	1
73	c.225C>G	p.L75L	3	0.49%(3/612)	0.06%(3/4704)	3
74	c.678T>C	p.A226A	6	0.16%(1/612)	0.02%(1/4704)	1
75	c.1905G>A	p.E635E	17	0.33%(2/612)	0.04%(2/4704)	2
76	c.2205T>G	p.S735S	19	0.16%(1/612)	0.02%(1/4704)	1
77	c.2283A>G	p.T761T	20	0.16%(1/612)	0.02%(1/4704)	1
78	2343+69 C>A		5′UTR	0.16%(**1**/612)	0.02%(1/4704)	1
79	intron7+(44_46)delACA		intron7	0.16%(1/612)	0.02%(1/4704)	1
80	intron9-(28_35)delTTTGTAGG		intron9	0.16%(1/612)	0.02%(1/4704)	1
81	Intron12-(7_13)insT		Intron12	0.16%(**1**/612)	0.02%(1/4704)	1
82	Intron18-56delCAAA		Intron18	0.82%(5/612)	0.11%(5/4704)	5
83	intron19-25T>A		intron19	0.49%(3/612)	0.06%(3/4704)	3
84	intron4-12T>A*		intron4			0
85	c.2217A>G*	p.Q739Q	19			0
86	c.2176A>G*	p.I726V	19			0

Notes:Variants No.1 to 83 were found in deaf patients.Variants No.84 and 86 labeled with * were carried by hearing normal controls;Mutational allele frequency: the number of mutant alleles/the number of total mutant alleles;FS:frameshift.

**Table 2:** Novel *SLC26A4* variants found in Chinese hearing loss population.

No.	Variants	Exon	Amino Acid	TM Domain
Variants in exons				
1	c.43_44insG	2	FS 15,stop at 86	NH2
2	c.68C>A	2	p.S23X	NH2
3	c.87G>C	2	p.E29D	NH2
4	c.234_235delC	3	FS79,stop at 96	NH2
5	c.249G>A	3	p.W83X	NH2
6	c.279T>A	3	p.S93R	NH2
7	c.665G>T	6	p.G222V	TM5
8	c.757A>G	6	p.I253V	EC LOOP 3
9	c.941C>T	8	p.S314L	TM7
10	c.1019_1020delT	9	FS 341,stop at 343	EC LOOP 4
11	c.1124A>G	9	p.Y375C	IC LOOP 4
12	c.1240G>A	10	p.E414K	EC LOOP 5
13	c.1245C>A	10	p.S415R	EC LOOP 5
14	c.1299_1300insC	11	FS 434,stop at 467	TM10
15	c.1327G>C	11	p.E443Q	IC LOOP 5
16	c.1409G>A	12	p.R470H	EC LOOP6
17	c.1472T>C	13	p.I491T	TM12
18	c.1517T>G	13	p.L506R	COOH
19	c.1595G>T	14	p.S532I	COOH
20	c.1645_1646insA	15	FS 549,stop at 563	COOH
21	c.1678G>A	15	p.D560H	COOH
22	c.1733_1735delATA	16	N579 lost, stop at 780	COOH
23	c.1897G>A	17	p.E633K	COOH
24	c.1979T>G	17	p.L660R	COOH
25	c.1983C>A	17	p.D661E	COOH
26	c.1985G>A	17	p.C662Y	COOH
27	c.1988G>A	17	p.G663E	COOH
28	c.1993A>G	17	p.I665V	COOH
29	c.2044G>T	18	p.E682X	COOH
30	c.2176A>G	19	p.I726V	COOH
31	c.2228T>A	19	p.L743X	COOH
32	c.2326C>G	21	p.R776G	COOH
**Variants in spicing site**				
33	IVS4+2T>C (415+2T>C)	intron4	Spicing site	
34	IVS13+5G>A (1544+5G>A)	intron13	Spicing site	
**Variant in UTR**				
35	2343+69C>A	21		
**Silent variants**				
36	c.225C>G	3	p.L75L	NH2
37	c.678T>C	6	p.A226A	TM5
38	c.1905G>A	17	p.E635E	COOH
39	c.2205T>G	19	p.S735S	COOH
40	c.2217A>G	19	p.Q739Q	COOH
41	c.2283A>G	20	p.T761T	COOH
**Variants in introns**				
42	intron4-12T>A	intron4		
43	intron7+(44_46delACA)	intron7		
44	intron9-(28_35)delTTTGTAGG	intron9		
45	intron 12-(7_13)insT	intron12		
46	intron18-(53_56)delCAAA	intron18		
47	intron19-25T>A	intron19		

FS:frameshift;X:stop codon;del:delete.

The mutational spectrum of *SLC26A4* differed among the various ethnicities. IVS7-2A>G was the most frequent hot-spot mutation in Han, Hui, Mongolian and minorities in the Southwest region, with an allele frequency of 8.01% (377/4704) and a mutant allele frequency of 61.6% (377/612). p.H723R was the second-most frequent hot-spot mutation in Han, Hui, and Mongolian ethnicities with an allele frequency of 1.51% (71/4704) and a mutant allele frequency of 11.6% (71/612). In contrast, neither IVS7-2A>G nor p.H723R were found in the Tibetan and Uygur patients.

The mutation detection rates were distinct in the hearing loss populations of different ethnicities, with the highest detection rate in the Han population (16.55%), followed by the Hui population (13.48%; homozygous mutation detection rate: 4.49%, compound heterozygous mutation detection rate: 3.37% and monoallelic mutation detection rate: 5.62%) and the Mongolian population (12.12%; homozygous mutation detection rate: 6.06%, compound heterozygous mutation detection rate: 0 and monoallelic mutation detection rate: 6.06%). In contrast, the mutation detection rate in the Tibetan population, and minorities in the Southwest region and Uygur, were low at 4.2, 2.4 and 1.45%, respectively.

The *SLC26A4* mutation detection rates differed geographically in China. Henan province ranked first with a detection rate of 31.58%. Detection rates in Gansu, Heibei, Hubei, Shaanxi and Heilongjiang province were 26.32, 25, 18.75, 18.37 and 16.67%, respectively. The *SLC26A4* mutation detection rate in Guangxi province, the Tibet Autonomous Region and Guizhou province were the lowest at 3.3, 5.08 and 5.4%, respectively. In Guangxi province, no biallelic *SLC26A4* mutations were found in the 90 patients enrolled. In the Tibet Autonomous Region, biallelic *SLC26A4* mutations were detected only in patients of Han ethnicity. The geographic distribution and the proportion of patients carrying two *SLC26A4* mutant alleles in each region are shown in Figure S1. Our results revealed that the incidence of EVA varied geographically in Mainland China. Surprisingly, we found that no Tibetan patients with EVA living in the Tibet plateau were diagnosed either by *SLC26A4* genetic testing or by high resolute temporal bone CT scan.

Four IVS7-2A>G heterozygotes, one missense c.2176A>G (p.I726V) heterozygote, one silent variant c.2217A>G (p.Q739Q), one silent variant c.225C>G (p.L75L) and one intron variant (intron4-12T>A) were found in the control group. Although this control population may be too small to reach a final conclusion, the carrier rate of *SLC26A4* mutations in China is estimated to be about 2.5%. Polymorphisms in *SLC26A4* appear to be rare in the general population in comparison to those in *GJB2* [22].

### Phenotype

Of the 342 hearing loss patients with mutations or variants in *SLC26A4*, 320 were examined by temporal CT scan or MRI. The follow-up of the remaining 22 patients (4 with biallelic mutations and 18 with monoallelic mutations) failed. All patients (240/240) with biallelic mutations or biallelic variants (not including silent variants) and 23.75% (19/80) of those with monoallelic mutations or variants in the ORF of *SLC26A4* were confirmed to have EVA by temporal bone CT scan or MRI (Figure S2). All patients diagnosed with EVA in our study showed moderate to profound bilateral sensorineural hearing impairment on audiograms (Figure S3). Because none of our patients showed goiter upon physical examination, the perchlorate discharge test was not performed in our study.

### Functional Study on the 10 SLC26A4 Variants

We chose 10 *SLC26A4* variants for functional study using the heterologous expression system. Of these, c.279T>A (p.S93R), c.665G>T (p.G222V), c.941C>T (p.S314L), c.1517T>G (p.L506R), c.1985G>A (p.C662Y), and c.2326C>G (p.R776G) have not been reported before. c.259G>T (p.D87Y), c.1079C>T (p.A360V), c.1225C>T (p.R409C) and c.1991C>T (p.A664V) have only been detected in ethnic Chinese [27]–[29]. Patients with the above 10 variants also carried an identified mutation, such as IVS7-2 A>G or c.2168 A>G (p.H723R) or c.1174 A>T (p.N392Y) on the other allele, and all were diagnosed with EVA by CT scan. The molecular epidemiological information and sift prediction of the pathogenicity of the ten variants are summarized in Table 3. The sift prediction of seven variants (p.D87Y, p.S93R, p.G222V, p.S314L, p.R409C, p.L506R, p.C662Y) was damaging, while the other three (p.A360V, p.A664V and p.R776G) was tolerated. All of the missense SLC26A4 variants, except p.L506R, p.C662Y and p.A664V, affect amino acids located in highly conserved regions of the *SLC26A4* orthologs. However, L506, C662 and A664 are located in conserved regions of most of the *SLC26A4* orthologs (Figure S4). None of the 10 *SLC26A4* variants was found in the normal-hearing control group.

**Table 3:** Molecular epidemiological information of ten *SLC26A4* variants in 2352 unrelated NSHL patients.

No	Variants	Exon	Amino Acid	Phenotype	Sift Prediction	Location in Pendrin	Variant frequency	Allele Frequency
1	c.259G>T	3	p.D87Y	EVA	DAMAGING	NH2	0.33%(2/612)	0.04%(2/4704)
2	c.279T>A	3	p.S93R	EVA	DAMAGING	TM1	0.33%(2/612)	0.04%(2/4704)
3	c.665G>T	6	p.G222V	EVA	DAMAGING	TM5	0.33%(2/612)	0.04%(2/4704)
4	c.941C>T	8	p.S314L	EVA	DAMAGING	TM7	0.16%(1/612)	0.02%(1/4704)
5	c.1079C>T	9	p.A360V	EVA	TOLERATED	TM8	0.16%(1/612)	0.02%(1/4704)
6	c.1225C>T	10	p.R409C	EVA	DAMAGING	ECLOOP 5	0.33%(2/612)	0.04%(2/4704)
7	c.1517T>G	13	p.L506R	EVA	DAMAGING	COOH	0.16%(1/612)	0.02%(1/4704)
8	c.1985G>A	17	p.C662Y	EVA	DAMAGING	COOH	0.16%(1/612)	0.04%(2/4704)
9	c.1991C>T	17	p.A664V	EVA	TOLERATED	COOH	0.16%(1/612)	0.02%(1/4704)
10	c.2326C>G	21	p.R776G	EVA	TOLERATED	COOH	0.16%(1/612)	0.02%(1/4704)

Membrane targeting is essential for the functional expression of a membrane-bound protein. We questioned whether these mutations could alter the folding of the 10 variants, which could result in reduced or loss of membrane expression. It has been demonstrated that pendrin shows robust membrane-targeted expression in HEK 293 cells. To determine whether these 10 variants were expressed in the membrane, we used confocal microscopy to examine the expression of EGFP and a membrane-based dye, di-8-amino-naphthylethenyl-pyridinium (di-8-ANEPPS) in HEK cells after transfecting the cells with plasmids containing one of the 10 variants tagged by EGFP. Di-8-ANEPPS is a member of the ANEP (aminoaphthyl ethenyl pyridinium) class of membrane potential dyes. Co-localization of EGFP expression and the di-8-ANEPPS dye in the membrane would suggest proper membrane targeting of the proteins [25]. Figure S5 shows representative images of membrane expression patterns of the 10 mutant pendrins. For comparison, membrane expression of the normal pendrin protein is also presented. Although it appeared that p.D87Y, p.S93R, p.G222V, p.R409C, p.L506R, p.C662Y, p.A664V and p.R776G all reached the plasma membrane, their expression levels were reduced when compared to normal pendrin. Other mutant pendrins (p.S314L, p.A360V, p.R409C, p.L506R, p.C662Y and p.R776G) were also localized to the cytosol, indicating retention in the intracellular region.

It has been suggested that the defect in Cl−/HCO_3_- exchange activities at the apical membrane of inner ear epithelial cells is the key factor that causes EVA and deafness [30]. We evaluated the transport activity of each mutant pendrin and compared it with that of WT-pendrin. A conventional radioisotope technique was used to measure [^14^C] formate uptake after sorting of EGFP-positive cells by fluorescence-based flow cytometry. Cells transfected with EGFP-vector only were used as a negative control. As shown in Figure S6, all mutant pendrins showed significantly decreased transport function compared to that of normal pendrin (*P*<0.01). Pfarr *et al*. reported that the p.R776C mutation does not compromise transporter activity [31]. We showed here that the transporter activity of p.R776G was significantly reduced, although the reduction was less dramatic than in other variants.

## Discussion

Mutations in *SLC26A4*, which encodes pendrin, are a common cause of deafness, and are responsible for both syndromic and non-syndromic hearing loss. The mutation spectrum of *SLC26A4* varies widely among ethnic groups [12]. In addition, the prevalence of mutations varies among ethnic groups. Campbell et al. reported p.T416P and IVS8+1G>A as the two most frequent mutations in northern European populations [6], whereas Blons et al. reported a completely different mutation spectrum that was extremely heterogeneous [13]. In a Japanese population, p.H723R accounted for 53% of the mutant alleles, and in a Korean population, p.H723R and the IVS7-2A>G mutation were the most prevalent, accounting for 45.5% of patients with PS or EVA [11], [20]. In China, the IVS7-2A>G mutation was the most common form, accounting for 57.63% of the mutant alleles [17].

All of the above studies focused on patients with EVA or Pendred syndrome. Few large-scale molecular epidemiological surveys on the *SLC26A4* gene in hearing loss populations have been reported. Previous studies verified that about 90% of Chinese patients with EVA carried at least one allelic mutation in exons 8, 19 or 10 of *SLC26A4* [17], [18], [23]. Because *SLC26A4* is a large gene with 20 exons, hot-spot region screening in a molecular epidemiological survey is an economical method. *SLC26A4* mutations were detected in nearly 15% of 2352 non-syndromic Chinese hearing impairment patients, with IVS7-2A>G being the most prevalent. By screening the above three exons of *SLC26A4*, we verified that the ratio of EVA in the Chinese deaf population was at least 11%, and that in the Han ethnicity reached at least 13%. Of our patients with EVA, 23.75% carried only one missense mutation. Whether the missense mutation causes a dominant negative effect and/or specifies a different phenotype is not clear. It is possible that the second mutant allele has not yet been identified due to the location of mutations deep in introns or promoter regions that were not sequenced, intragenic exon deletions, or the involvement of mutations in genes other than *SLC26A4*, such as *FOXI* and *KCNJ10* (i.e., digenic synergistic mutations). However, none of our patients with one *SLC26A4* missense mutation carried any of the reported *FOXI* or *KCNJ10* mutations.

We found that different ethnicities that originated from various regions of China showed distinct *SLC26A4* mutational spectra and detection rates, which may be explained by the genetic heterogeneity in various ethnicities. For example, the mutation detection rates in the Tibetan population and minorities in the Southwest region and Uygur populations were significantly lower than that in the Han, Hui and Mongolian populations. Although IVS7-2A>G and p.H723R were hot mutations in Han, Hui and Mongolian patients, they were not found in the Tibetan and Uygur patients. Our results also revealed that the *SLC26A4* detection rate and incidence of EVA varied geographically. The incidence of inner ear malformation in the Tibetans living in the Tibet plateau was almost as high as that in Chinese Han patients, but the types of malformation in the Tibetan population differed greatly [32]. The most-common inner ear deformity, EVA, is rare in the Chinese Tibetan hearing-loss population. Hypoxia may be one of the causes of development of inner ear deformity, but further studies are required to determine the genetic etiology of hearing loss in Tibetan patients. We also found that the detection rate of another common gene, *GJB2*, was significantly lower in Guangxi province (6.05%) than the average rate in China (21.01%) [33]. These results may be explained by the influence of regional or environmental on the etiology of hearing loss in Guangxi.

A striking achievement of this study is the establishment of a new strategy for detecting *SLC26A4* mutations prior to temporal bone CT scan for identifying patients with EVA. In China, the cost of a temporal CT scan is 200 to 300 RMB (approximately 3 days’ salary of an average Chinese). Because of the relatively high cost, it is not possible to perform a CT scan in every hearing loss patient for diagnosis of EVA. Because 97.9% of Chinese patients with EVA carry an *SLC26A4* mutation [17], *SLC26A4* mutations in hearing loss patients indicate a high possibility of EVA. This model has the unique advantage of identifying patients with EVA in an epidemiologic study in large-scale deaf populations.

The discovery of 47 novel variants enriches the mutational spectrum of *SLC26A4*. Functional tests of mutated pendrin allelic variants found in patients with Pendred syndrome or non-syndromic EVA revealed that the pathology is linked to a reduction or a loss of function in the ion transport activity of pendrin [34], [35]. Subcellular localization studies revealed that the mutated proteins are often retained in subcellular compartments and are unable to reach the plasma membrane [36], [37]. These mutations most likely cause protein misfolding, followed by impaired trafficking and subsequent degradation. Furthermore, the transport function of mutated proteins that do reach the membrane is impaired [34], [38], [39]. Besides clinical and radiological assessments, the functional evaluation of pendrin mutations is essential for correct diagnosis of Pendred syndrome and non-syndromic EVA [40]. Indeed, the clinical condition of pseudo-Pendred syndromes [41]–[43] and the high incidence of functional pendrin polymorphisms in some populations [30] could lead to an incorrect assignment of the pathological conditions in cases where proper functional characterizations are not possible. Whereas a broad spectrum of mutations is found in Caucasian populations, and Israeli and Palestinian populations have been functionally characterized [44], [45], little about the variants that occur in Chinese is known.

We used confocal microscopy and radioisotope techniques to examine the membrane expression and transporter function of 10 mutant proteins in human embryonic kidney cells. We showed that all 10 mutations resulted in a significant reduction in membrane expression of the proteins. Measurement using a radioisotope technique showed that their transport activity was significantly reduced. Our results suggest that the pathogenesis underlying the hearing loss associated with these mutations is the result of reduced membrane expression and decreased transport capability of the proteins.

Pera et al. [30] suggested that certain biochemical parameters explained some functional impairment. For example, transport function was impaired if an amino acid bearing a fixed charge was missing or introduced (i.e., aspartic acid (D) with fixed negative charge (K) or glutamic acid (EK), lysine (K) with positive fixed charge (C) or arginine (RC), and histidine (HC) with the positive charge of histidine, depending on the pH). Furthermore, the loss or inclusion of a proline (P; proline acts as a structural disruptor of regular secondary structures such as α-helices or β-sheets) in the *SLC26A4* sequence is detrimental to its transport function. However, Pfarr et al. [31] described p.R776C as an exception, with an arginine located on the extreme C-terminus of *SLC26A4* (a 780 amino acid protein). It is thought that the N- and C-termini of a protein are not structural and the mutations occurring in these areas probably have little or no functional impact [30]. However, with regard to the EVA phenotype, the p.R776G mutation in Chinese was found at very low allele frequency in the control group.

In conclusion, we provide an overview of the molecular epidemiological status of *SLC26A4* in China. We showed that the prevalence of EVA in the Chinese deaf population was at least 11%, which reached at least 13% in the Han majority population. The mutational spectrum and mutation detection rate of *SLC26A4* are distinct among both ethnicities and regions of mainland China. Our results suggest that the pathogenesis underlying hearing loss in patients with EVA is the result of reduced membrane expression and the decreased transport activity of the mutant pendrins. The functional assessment procedure can be applied to genetic diagnosis of EVA patients and prenatal diagnosis for their families. These findings are valuable for genetic counseling and risk assessment for families with EVA in China.

## Supporting Information

Figure S1 **Geographic distribution and the proportion of patients carrying two** ***SLC26A4*** **mutant alleles in each region studied.** NE: not examined
(TIF)

Figure S2 **Examples of CT and MRI images of normal children and children with** ***SLC26A4*** **mutations.** A: CT of temporal bone of a normal child. Arrows show the normal vestibular aquaduct. B: CT of a patient with EVA. Arrows mark the enlarged vestibular aquaduct. C: MRI of child with normal inner ear. Arrows indicate the normal endolymphatic sac. D: MRI of a patient with EVA. Arrows mark the hydrop of endolymphatic sac.
(TIF)

Figure S3 **Representative pure-tone audiograms of children with** ***SLC26A4*** **mutations.** A: c.259G>T/c.2168A>G. B: c.941C>T/c.1174A>G. C: c.1517T>G/IVS7-2A>G. D: c.1991C>T/IVS7-2A>G
(TIF)

Figure S4 **Protein sequences of 13 SLC26A4 orthologs, including: (1) Homo sapiens (NP_000432.1), (2) Nomascus leucogenys (XP_003268184.1), (3) Pan troglodytes (XP_519308.2), (4) Macaca mulatta (XP_001094049.1), (5) Callithrix jacchus (XP_002751785.1), (6) Sus scrofa (XP_003357559.1), (7) Canis lupus familiaris (XP_540382.3), (8) Rattus norvegicus (NP_062087.1), (9) Mus musculus (NP_035997.1), (10) Oryctolagus cuniculus (XP_002712085.1), (11) Loxodonta africana (XP_003407255.1), (12) Xenopus (Silurana) tropicalis (NP_001107135.1), and (13) Monodelphis domestica (XP_001363598.1).**
(TIF)

Figure S5 **Heterologous expression of wild-type and mutant pendrins.** HEK cells were transfected with each plasmid of the 10 variants tagged by EGFP. A membrane-based dye (orange color), di-8-ANEPPS, was also added in the solution before confocal microscopy. Co-localization of EGFP expression and the di-8-ANEPPS dye in the membrane would suggest proper membrane targeting of the proteins.
(TIF)

Figure S6 **Transport activity of wild-type pendrin and 10 mutant pendrins.**
(TIF)

Methods S1 **SLC26A4 Site-Directed Mutagenesis Primers.** The mRNA from normal human adenoid specimens was extracted, and cDNA was synthesized. PCR was done to catch the target gene. Primers were as follows: *Xho*I -F:5′- CCGCTCGAGATGGCAGCGCCAGGCGGCAG -3′ KpnI -R:5′- GGGGTACCGTGGATGCAAGTGTACGCATAG -3′ *Xho*I -F contains protective base and XhoI restriction sites, its 5 'end partial sequence for PCR amplify target gene. KpnI -R contains protective base and KpnI restriction sites,its 3 'end partial sequence for PCR amplify target gene. The entire ORF of the human SLC26A4 gene was cloned into the expression vector pEGFP-N1 (Invitrogen, Carlsbad, CA, USA). The point mutation-specific primers were synthesized as shown in Table S1.
(DOCX)

Table S1 **SLC26A4 Site-Directed Mutation Primers.**
(DOCX)
