# Supplementary figures and images for: Molecular Epidemiology and Functional Assessment of Novel Allelic Variants of SLC26A4 in Non-Syndromic Hearing Loss Patients with Enlarged Vestibular Aqueduct in China
Source: PLoS One. 2012 Nov 21;7(11):e49984. doi: 10.1371/journal.pone.0049984 (PMC3503781; doi:10.1371/journal.pone.0049984)

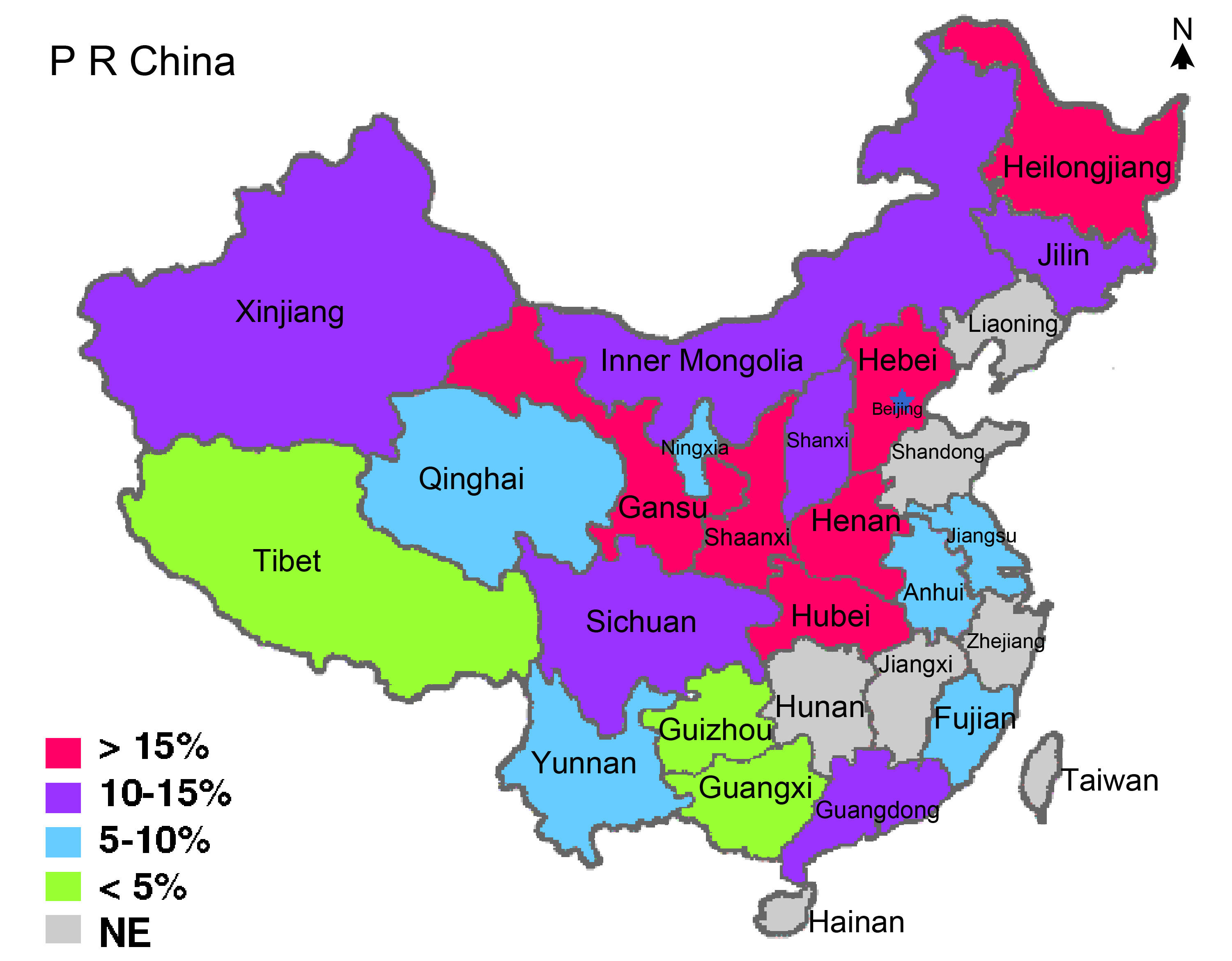

Supplement: Figure S1 — Geographic distribution and the proportion of patients carrying two SLC26A4 mutant alleles in each region studied. NE: not examined (TIF) [file pone.0049984.s001.tif]

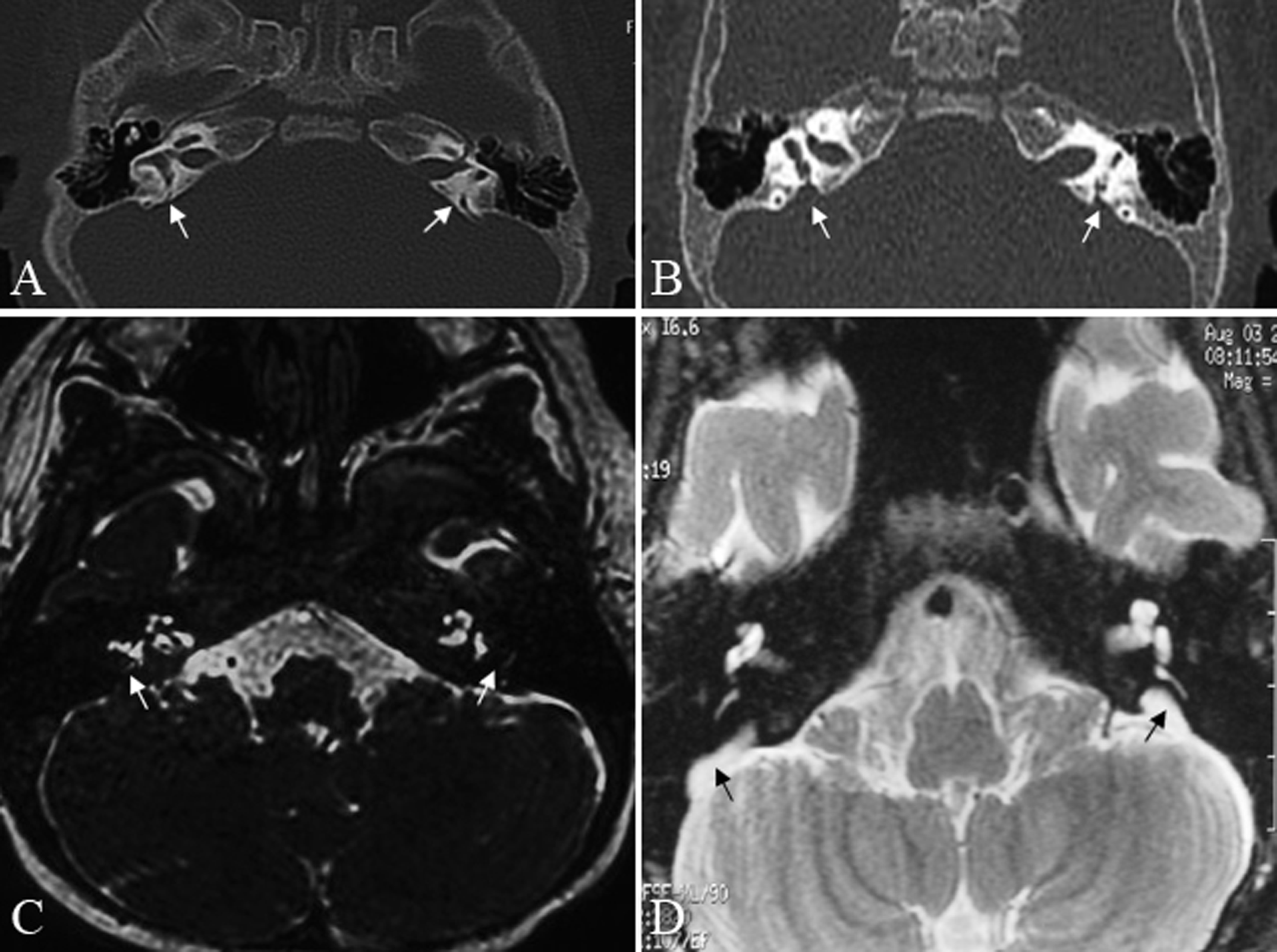

Supplement: Figure S2 — Examples of CT and MRI images of normal children and children with SLC26A4 mutations. A: CT of temporal bone of a normal child. Arrows show the normal vestibular aquaduct. B: CT of a patient with EVA. Arrows mark the enlarged vestibular aquaduct. C: MRI of child with normal inner ear. Arrows indicate the normal endolymphatic sac. D: MRI of a patient with EVA. Arrows mark the hydrop of endolymphatic sac. (TIF) [file pone.0049984.s002.tif]

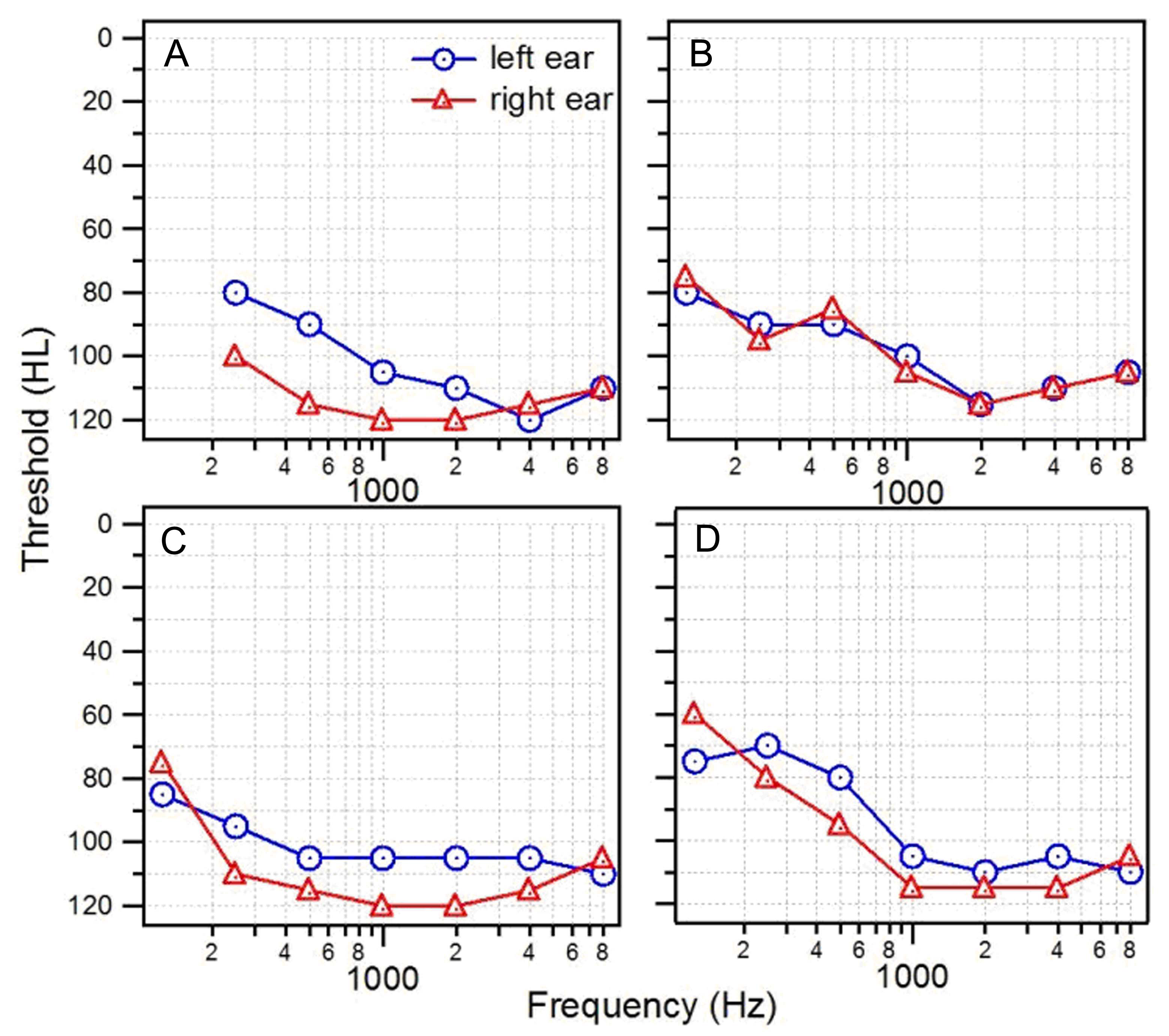

Supplement: Figure S3 — Representative pure-tone audiograms of children with SLC26A4 mutations. A: c.259G>T/c.2168A>G. B: c.941C>T/c.1174A>G. C: c.1517T>G/IVS7-2A>G. D: c.1991C>T/IVS7-2A>G (TIF) [file pone.0049984.s003.tif]

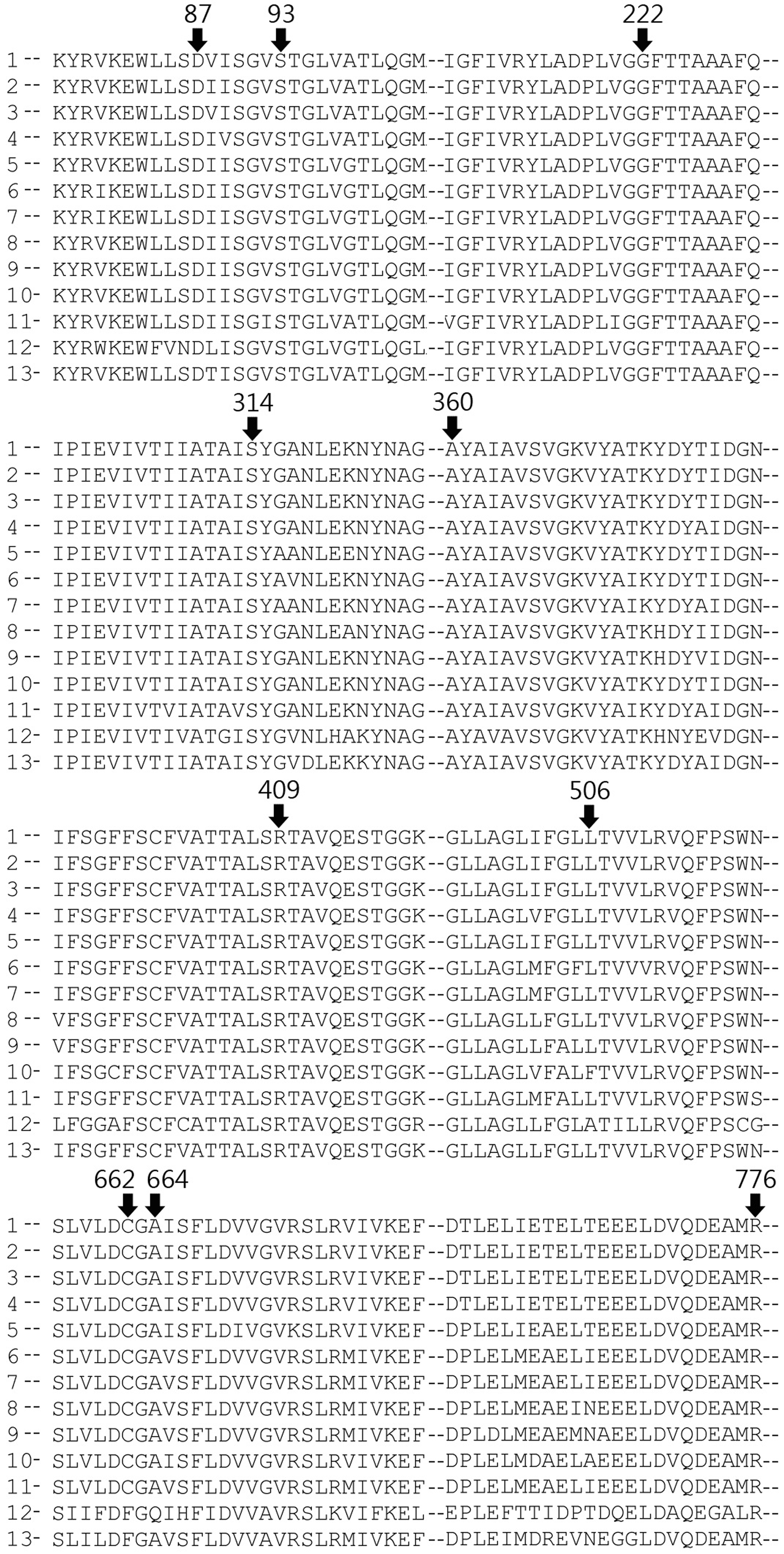

Supplement: Figure S4 — Protein sequences of 13 SLC26A4 orthologs, including: (1) Homo sapiens (NP_000432.1), (2) Nomascus leucogenys (XP_003268184.1), (3) Pan troglodytes (XP_519308.2), (4) Macaca mulatta (XP_001094049.1), (5) Callithrix jacchus (XP_002751785.1), (6) Sus scrofa (XP_003357559.1), (7) Canis lupus familiaris (XP_540382.3), (8) Rattus norvegicus (NP_062087.1), (9) Mus musculus (NP_035997.1), (10) Oryctolagus cuniculus (XP_002712085.1), (11) Loxodonta africana (XP_003407255.1), (12) Xenopus (Silurana) tropicalis (NP_001107135.1), and (13) Monodelphis domestica (XP_001363598.1). (TIF) [file pone.0049984.s004.tif]

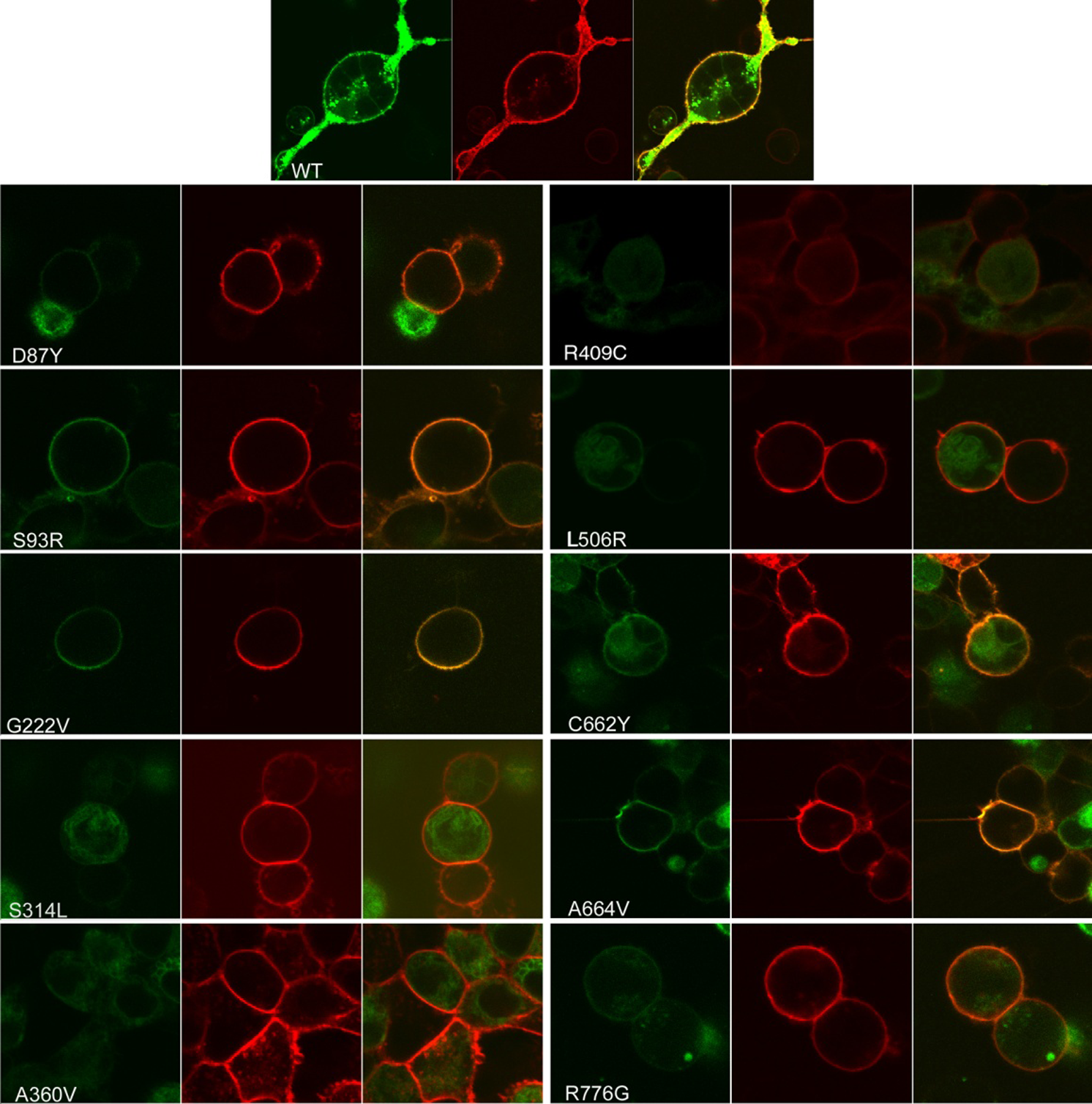

Supplement: Figure S5 — Heterologous expression of wild-type and mutant pendrins. HEK cells were transfected with each plasmid of the 10 variants tagged by EGFP. A membrane-based dye (orange color), di-8-ANEPPS, was also added in the solution before confocal microscopy. Co-localization of EGFP expression and the di-8-ANEPPS dye in the membrane would suggest proper membrane targeting of the proteins. (TIF) [file pone.0049984.s005.tif]

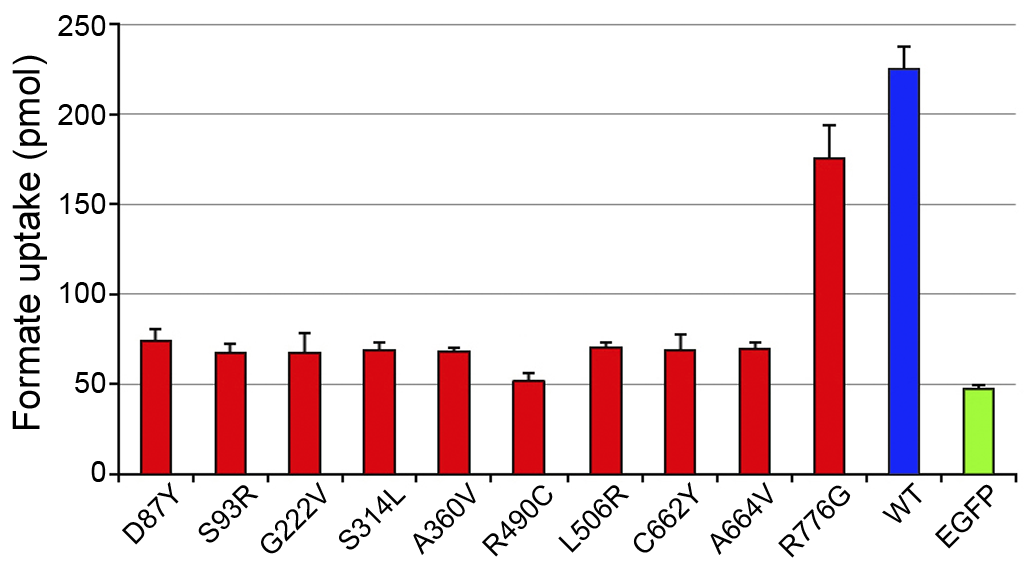

Supplement: Figure S6 — Transport activity of wild-type pendrin and 10 mutant pendrins. (TIF) [file pone.0049984.s006.tif]
