# Supplementary material for: Molecular Epidemiology and Functional Assessment of Novel Allelic Variants of SLC26A4 in Non-Syndromic Hearing Loss Patients with Enlarged Vestibular Aqueduct in China
Source: PLoS One. 2012 Nov 21;7(11):e49984. doi: 10.1371/journal.pone.0049984 (PMC3503781; doi:10.1371/journal.pone.0049984)
Supplement: Methods S1 — SLC26A4 Site-Directed Mutagenesis Primers. The mRNA from normal human adenoid specimens was extracted, and cDNA was synthesized. PCR was done to catch the target gene. Primers were as follows: XhoI -F:5′- CCGCTCGAGATGGCAGCGCCAGGCGGCAG -3′ KpnI -R:5′- GGGGTACCGTGGATGCAAGTGTACGCATAG -3′ XhoI -F contains protective base and XhoI restriction sites, its 5 'end partial sequence for PCR amplify target gene. KpnI -R contains protective base and KpnI restriction sites,its 3 'end partial sequence for PCR amplify target gene. The entire ORF of the human SLC26A4 gene was cloned into the expression vector pEGFP-N1 (Invitrogen, Carlsbad, CA, USA). The point mutation-specific primers were synthesized as shown in Table S1. (DOCX) [file pone.0049984.s007.docx]

**SLC26A4 Site-Directed Mutagenesis Primers**

The mRNA from normal human adenoid specimens was extracted, and cDNA was synthesized. PCR was done to catch the target gene . Primers were as follows:
*Xho*I -F：5’- CCGCTCGAGATGGCAGCGCCAGGCGGCAG -3’

KpnI -R：5’- GGGGTACCGTGGATGCAAGTGTACGCATAG -3’

*Xho*I -F contains protective base and XhoI restriction sites, its 5 'end partial sequence for PCR amplify target gene. KpnI -R contains protective base and KpnI restriction sites,its 3 'end partial sequence for PCR amplify target gene. The entire ORF of the human SLC26A4 gene was cloned into the expression vector pEGFP-N1 (Invitrogen, [Carlsbad, CA, USA](file:///C:\Users\yyymzh\AppData\Local\Temp\AppData\Local\Microsoft\wiki\Carlsbad,_California)).

**SLC26A4 Site-Directed Mutagenesis Primers**

The point mutation-specific primers were synthesized as shown in Table S1.

**Table S1. SLC26A4 Site-Directed Mutation Primers**

| Primer Name | Sequence (5’-3’) | Tm(C°) |
| --- | --- | --- |
| SLC26A4- D87Y -F | tacgtcatttcgggagttag | 55 |
| SLC26A4- D87Y -R | actaagcagccattccttga | 55 |
| SLC26A4 - S93R -F | aactgggctagtggccacgct | 55 |
| SLC26A4- S93R -R | ctaactcccgaaatgacg | 55 |
| SLC26A4- G222V -F | tcttcacaacagctgct | 55 |
| SLC26A4- G222V -R | agctatatctttcctta | 55 |
| SLC26A4- S314L -F | tatatggagccaacctggaa | 55 |
| SLC26A4- S314L -R | aaatggcagtagcaattatcgtc | 55 |
| SLC26A4- A360V -F | tttatgctattgcagtgtcag | 55 |
| SLC26A4- A360V -R | ccaccacagcgatggaaaatg | 55 |
| SLC26A4- R409C -F | tgcacggccgtccagga | 55 |
| SLC26A4- R409C -R | cacagtcaagcacaag | 55 |
| SLC26A4- L506R -F | ggttgactgtggtcctgaga | 55 |
| SLC26A4- L506R -R | gtccaaatataaggccagcta | 55 |
| SLC26A4- C662Y -F | atggagctatatctttcctg | 55 |
| SLC26A4- C662Y -R | agtcaagcacaaggctatgga | 55 |
| SLC26A4- A664V -F | ttatatctttcctggacgttg | 55 |
| SLC26A4- A664V -R | ctccacagtcaagcacaagg | 55 |
| SLC26A4- R776G -F | ggtacacttgcatcct | 55 |
| SLC26A4- R776G -R | catagcctcatcctggacatc | 55 |
